# Metabolic syndrome in perimenopausal and postmenopausal women attending gynaecology outpatient department at a tertiary institution in South Africa

**DOI:** 10.1016/j.xagr.2025.100560

**Published:** 2025-08-16

**Authors:** Lawrence Marapo, Lineo Matsela, ME Chabalala, Olakunle Adewunmi Towobola

**Affiliations:** a Department of Obstetrics and Gynecology, Sefako Makgatho University, Pretoria, Gauteng, South Africa; b Department of Internal Medicine and Clinical research, Sefako Makgatho University, Pretoria, Gauteng, South Africa

**Keywords:** dyslipidemia, glucose, hormonal profile, metabolic syndrome, outpatient department, perimenopausal, postmenopausal women, prevalence, waist circumference, vasomotor symptoms

## Abstract

**Background:**

The prevalence of metabolic syndrome in menopause varies globally. There is a paucity of data regarding knowledge of metabolic syndrome in menopause in South Africa.

**Objective(s):**

The study was designed to describe the prevalence of metabolic syndrome in perimenopausal and postmenopausal and the lipid changes that occur during the menopause transition.

**Study Design:**

A prospective cross-sectional study was conducted at Dr George Mukhari Academic Hospital, the gynaecological outpatient department, Pretoria, South Africa. Perimenopausal and postmenopausal women were included. Six hundred ninety menopausal women were enrolled in this study. The participants were evaluated by physical examination. Blood samples were also taken for biochemical assay. The researcher also took their blood pressure. Data compiled from the study were analyzed using the Statistical Package for Social Sciences version 21. Descriptive statistical analysis established the range, mean (including 95% confidence interval), and standard deviation for quantitative variables. Categorical variables were analysed using Fisher's exact test, and risk analysis of factors associated with Metabolic syndrome was performed by calculating the odds ratio (OR) and its 95% confidence interval for both perimenopausal and postmenopausal women. A 2-tailed statistical analysis yielding a p-value <.05 was taken as statistically significant.

**Results:**

The study consisted of 690 women (n=690), including 338 perimenopausal women (n=338; 49.0%) and 352 postmenopausal women (n=352; 51.0%). The prevalence of metabolic syndrome was noted to be 47% and 61.4% in the perimenopausal and postmenopausal groups, respectively. The mean waist circumference was higher than the cut-off value of 88cm for perimenopausal and postmenopausal women. There were no statistically significant differences in lipid profiles between perimenopausal and postmenopausal women. The odds ratio for elevated triglycerides was 0.65 (95% CI: 0.32–1.01) and for low HDL levels was 0.83 (95% CI: 0.41–0.98). Perimenopausal women had significantly higher odds (OR 2.19, *P<.*0001) of experiencing hot flushes compared to postmenopausal women. Multivariate logistic regression analysis demonstrated that age (*P=.*3789) was not significantly associated with metabolic syndrome. However, the prevalence of metabolic syndrome was higher in postmenopausal women compared to perimenopausal women. Sleep disorders (*P<.*0001) and vasomotor symptoms (*P=.*0198) showed significant correlations with the presence of metabolic syndrome. When comparing women with and without metabolic syndrome, both perimenopausal (HDL; *P<.*0001, triglycerides; *P<.*0001) and postmenopausal groups (HDL; *P<.*0001, triglycerides; *P<.*0001) demonstrated significant associations between dyslipidaemia and metabolic syndrome.

**Conclusion:**

This study reflects the current high burden of metabolic syndrome amongst perimenopausal and postmenopausal women in South Africa. The prevalence of metabolic syndrome was noted to be higher in postmenopausal women as compared to perimenopausal women. There was a correlation between metabolic syndrome and age, sleep disorders, vasomotor symptoms, and dyslipidaemias.

**AJOG Global Reports at a Glance**

**A. Why was this study conducted?**

The study focused on the prevalence of metabolic syndrome in menopause and the lipid changes highlighted during this transition. The main reason for the study was to magnify the burden of metabolic syndrome in menopause in South Africa.

**B. What are the key findings?**

In this prospective cross-sectional study, the prevalence of metabolic syndrome was noted to be 47% and 61.4% in perimenopausal and postmenopausal women, respectively. There was a strong correlation between age and metabolic syndrome. There was homogeneity in comparing the lipid profiles between perimenopausal and postmenopausal women. Perimenopausal women had significantly higher odds (OR 2.19, *P<.*0001) of experiencing hot flushes compared to postmenopausal women.

**C. What does this study add to what is already known?**

This study demonstrated that menopause on its own is a moderate to high risk factor for metabolic syndrome. The study finding reflects the current high burden of metabolic syndrome amongst perimenopausal and postmenopausal women in South Africa.

## Introduction

Metabolic syndrome refers to the co-occurrence of several known cardiovascular risk factors, including insulin resistance, central obesity, atherogenic dyslipidaemia, and hypertension.^7^ Other causative factors include physical inactivity, aging, and hormonal imbalance, such as polycystic ovarian syndrome and menopause.

According to Seong-Hee Ko and co-workers^2^ menopause is the cessation of menstruation for 1 year due to the loss of ovarian follicular activity. It typically occurs at around 45–55 years of age. In the USA, the average menopause age is 51. In South Africa, the average age of menopause is 51.6.^5^

Menopausal transition, is an interval during which many women have irregular menstrual cycles. Early menopause transition, in which menstrual lengthening of up to 7 days occurs before menopause. Late menopause transition presents as episodes of amenorrhoea lasting at least 3 months until final menstruation period. Postmenopausal is defined as a period that starts after amenorrhoea lasting 12 months. Perimenopause encompasses early and late menopause transition and early postmenopausal stage 1a.^6^

The study focused on the prevalence of metabolic syndrome in menopause and the lipid changes highlighted during this transition. The main reason for the study was to magnify the burden of metabolic syndrome in menopause in South Africa.

There is a noticeable gap in the knowledge of metabolic syndrome in menopause in South Africa (Chikwati R.P et al., 2024). As patients transition from perimenopause to menopause, there is an accumulation of adipose tissue with the consequence of abdominal obesity.^8^ Metabolic syndrome increases the risk of both diabetes and cardiovascular diseases, resulting in poor quality of life in the affected women.^9^ The development of metabolic syndrome affects the women financially, physically, and emotionally as they will incur more costs to ensure that they are medically viable. The life expectancy of women in South Africa is 63 years old, while in America, 82 years old, according to the United Nations Population Division.^10^ Life expectancy differences imply an improved understanding of metabolic syndrome in menopause.

According to Seong-Hee Ko and co-workers,^2^ the prevalence of metabolic syndrome in menopause is higher in developed countries than in developing countries because metabolic syndrome is linked to diet and lifestyle.

The prevalence of obesity, including in women, is increasing globally, and it is a poorly studied and neglected concept. Jennifer L and co-workers^1^ confirmed that cardiovascular disease, which is a complication of metabolic disease, kills more women than men.

This study aimed to raise awareness and provide a comprehensive understanding of how metabolic syndrome impacts African women, thereby improving their quality of life. The menopausal transition is associated with increased adiposity and an accelerated risk of cardiovascular events due to hormonal fluctuations and lipid alterations, as highlighted by Collins et al. (2017). In South Africa, a developing country undergoing rapid urbanisation, the adoption of sedentary Western lifestyles and the increased consumption of processed and fast foods have contributed to rising rates of obesity. It is therefore plausible to anticipate that metabolic syndrome during menopause will become a significant global health burden, with developing countries being disproportionately affected. At our institution, the majority of menopausal women present with obesity, underscoring the need to examine the relationship between menopausal transition and metabolic risk factors. This concern necessitated an investigation into the prevalence of metabolic syndrome and associated lipid changes during menopause, to guide healthcare professionals in Africa towards establishing targeted protocols for the management of menopausal women with metabolic syndrome.

There is scarcity of literature on this subject in South Africa. A study done by Gradidge PJL and co-workers^12^, noted the prevalence of metabolic syndrome in black South African women to be 42%, this study included women in the reproduction age.

Mesch and co-workers (2013) observed the influence of age versus menopause on metabolic syndrome, which has shown that postmenopausal women had a 20–22% chance of having metabolic syndrome as compared to 0% in premenopausal women.

A study conducted in Poland, as highlighted by Dorotha and co-workers (2017), aimed to analyse the frequency of metabolic syndrome in perimenopausal and postmenopausal women who are doing intellectual work. The same study found that the prevalence of metabolic syndrome and its criteria did not depend on demographic variables. The study concluded that the prevalence is related to clinical characteristics and not intellectual work. The prevalence of metabolic syndrome in perimenopause was 14.33% as compared to 52.33% recorded in postmenopausal women. The study done by Jeyasheela K and co-workers,^3^ which assessed the prevalence of metabolic syndrome (MetS) among 154 postmenopausal women in South India, was comparable, revealing a significant prevalence rate of 64%. The study by Zahra J and co-workers (2013), which assessed the prevalence of metabolic syndrome (MetS) among 118 postmenopausal women in Tehran, found contrasting results, revealing a 30.1% prevalence rate.

The most consistent lipid alteration during menopause is a rise in LDL. The SWAN study and the Framingham Offspring Study both demonstrated a 10–20% increase in LDL in the first year after the final menstrual period (Khoudary et al., 2016). Mechanistically, estrogen withdrawal reduces LDL receptor activity, leading to impaired clearance of LDL particles and increased hepatic cholesterol synthesis. Elevated LDL-C in postmenopausal women is strongly correlated with subclinical atherosclerosis and CVD incidence (Khoudary et al., 2016).

### Diagnosis

#### Menopause transition

The universal diagnosis of perimenopausal and postmenopausal women is by STRAW + 10, which emphasizes clinical diagnosis over biochemical diagnosis. In contrast, a study done by Bertone-Johnson and co-workers (2018) suggested that AMH could be used as a clinical marker in diagnosing early perimenopause. The studies suggesting AMH as a diagnostic criterion for early perimenopause are inadequate. Consequently, clinical diagnosis of menopause transition by STRAW + 10 remains the gold standard.

#### Metabolic syndrome

The National Cholesterol Education Program (NCEP) Adult Treatment Plan III devised the definition of metabolic syndrome in 2001, which was last updated by the American Heart Association in 2005.^11^

NCEP ATP III confirms the diagnosis of metabolic syndrome if 3 or more of the following 5 criteria are present.

1. Waist circumference >88 cm in women
2. BP >130/85
3. Fasting TG >1.69 mmol/L
4. Fasting HDL cholesterol <1.29 mmol/L
5. Fasting glucose >5.6 mmol/L

The objectives of this study were:

1. To determine the prevalence of metabolic syndrome in perimenopausal and postmenopausal women in Dr George Mukhari Academic Hospital gynaecology outpatient department.
2. To analyse the lipid profile changes during the menopause transition.

## Materials and methods

A descriptive cross-sectional study was conducted from March 2022 to March 2025. The study was conducted at the Outpatient Gynaecology Department of Obstetrics and Gynaecology of Dr George Mukhari Academic Hospital, in Ga-Rankuwa (Tshwane district of Gauteng Province), Pretoria, South Africa. This tertiary-level hospital is attached to Sefako Makgatho Health Sciences University. The target population included women who were in the perimenopausal and postmenopausal stages. Perimenopausal or Postmenopausal women who had hysterectomies, with defined and advanced gynaecological malignancies, who were on menopause hormonal therapies, and with a documented cause of abnormal uterine bleeding and a documented cause of secondary amenorrhoea were excluded from the study. This study used a consecutive sampling method until the required sample size of 690 was reached, from March 2022 to March 2025.

Informed consent to participate in the study was obtained. On their first visit, the participants were evaluated by physical examination. The researcher took their mass and height to calculate their Body mass index (BMI), which was calculated using the Du Bois formula (mass (kg)/height (m)^2^). Blood samples were also taken for biochemical and hormonal assay. The researcher also took their blood pressure. Participants were requested to be reviewed for fasting blood glucose levels. Waist circumference was measured.

Blood pressure was measured using the Drager BP machine, which the researcher calibrated every morning. Mass and height were measured using the Seca mass and height machine, which the researcher also calibrated daily. Fasting glucose was taken using the accu-check glucose meter. Blood for biochemical assays was collected using a 5 ml syringe, green-topped needle, gray-topped, yellow-topped, and purple-topped vacutainer tubes.

Data compiled from the study was subjected to statistical analysis using the Statistical Package for Social Sciences (SPSS) version 21. Descriptive statistical analysis established the range, mean (including 95% confidence interval), and standard deviation for quantitative variables. Categorical variables were analysed using Fisher's exact test, and risk analysis of factors associated with MetS was performed by calculating the odds ratio (OR) and its 95% confidence interval for both perimenopausal and postmenopausal women. A 2-tailed statistical analysis yielding a p-value <.05 was taken as statistically significant.

### Ethical consideration

Before the study commenced, the Ethics Committee of Sefako Makgatho Health Sciences University granted institutional ethics approval after reviewing the study protocol: (Certificate Number: SMUREC/M/15/2022: PG). The clinical manager of Dr George Mukhari Academic Hospital also granted permission to conduct the study. Confidentiality and anonymity were maintained throughout the study. Informed consent was obtained from every patient prior to conducting the study.

### Results

The study consisted of 6 hundred and ninety women (n=690), made up of 3 hundred and thirty-eight (n=338; 49.0%) perimenopausal women and 3 hundred and fifty-two (n=352; 51.0%) postmenopausal women. The study data have been calculated for range, mean, and standard deviation (SD), and where necessary, data were also calculated for median and interquartile range. Differences between the 2 study groups (perimenopausal and postmenopausal women) were calculated for statistical significance at a threshold for p-value of ≤.05).

Table 1 show the demographic features of the women using the stratified study components of perimenopausal and postmenopausal. There was a statistically significant difference (*P<.*0001) in the age pattern of the women: perimenopausal women (mean=46.5 yrs.; SD=3.8 yrs.) versus postmenopausal women (mean=55.6 yrs.; SD=8.2 yrs.). Most participants were black Africans; in terms of parity patterns, there was no significant statistical difference between the 2 groups. There was a statistically significant difference in unemployment (*P=.*0113), employment (*P=.*0350), and pensioners (0.0028) in terms of employment status between the 2 groups. Self-employment yielded no significant statistical difference between the 2 groups.

**Table 1:** Demographic characteristics of the women (peri- versus post-menopause)

Demographics		Perimenopause (n=338]	Post-menopause (n=352]	*P-value*
Age (yrs):				
	Range	39–54	49–79	
	Mean	46.5	55.6	<.0001
	SD	3.8	8.2	
Parity:	Range	0–7	0–8	
	*para: 0*	17 (5.0%)	12 (3.4%)	-
	*para: 1–3*	216 (64.1%)	183 (52.0%)	*.1285*
	*para: 4–6*	95 (28.2%)	124 (35.2%)	*.4622*
	*para: >6*	9 (2.7%)	33 (9.4%)	*.1186*
Race:				
	Black Africans	334 (98.8%)	352 (100.0%)	*-*
	Coloured	1 (0.3%)	-	*-*
	White	3 (0.9%)	-	*-*
Employment status				
Unemployed		163 (48.2%)	113 (32.1%)	*.0113*
Employed		122 (36.1%)	74 (21.0%)	*.0350*
Pensioners		16 (4.7%)	84 (23.9%)	*.0028*
Self-employed		18 (5.3%)	25 (7.1%)	*.3410-*
Unknown		19 (5.6%)	56 (15.9%)	

**Figure 1:**
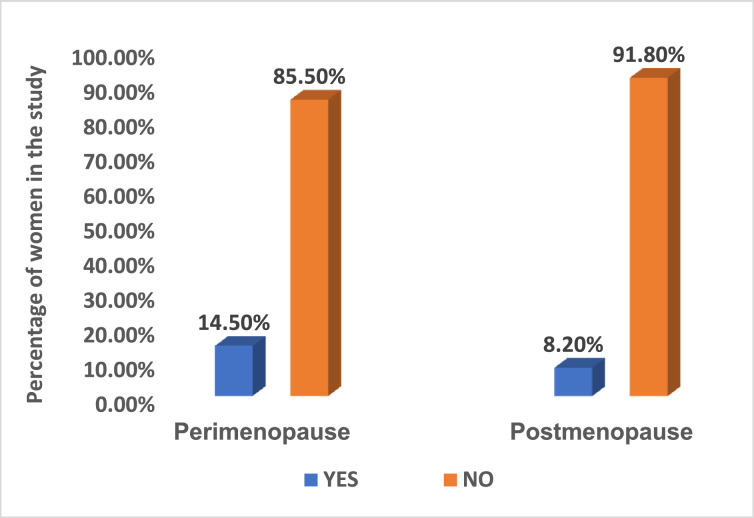
Smoking habits of peri- & postmenopausal women in this study.

Figure 1 illustrates the smoking habits of the women in the study. There was no statistically significant difference in the smoking habits between perimenopausal (n=49; 14.5%) and postmenopausal women (n=29; 8.2%) with a p-value of .3682.

Marapo. Metabolic syndrome in perimenopausal and postmenopausal women. Am J Obstet Gynecol 2025.

Table 2 shows the differences in psycho-somatic symptoms between the perimenopausal and postmenopausal women. Among the psycho-somatic symptoms assessed, perimenopausal women had significantly higher odds of experiencing hot flushes (OR 2.19, *P<.*0001) compared to postmenopausal women; these differences were statistically significant. Although higher odds were observed for heart palpitations (OR 1.55, *P=.*3826), sleep disorders (OR 1.67, *P=.*0886), and headaches (OR 1.17, *P=.*2644) among perimenopausal women, these differences were not statistically significant. The odds of irritability (OR 1.06, *P=.*4852) and joint pain (OR 1.06, *P=.*7363) were similar between groups; these differences were also not statistically significant.

**Table 2:** Psycho-somatic symptoms of peri- (n=338) and postmenopausal women (n=352)

Symptoms	Perimenopause (No & [%])	Post-menopause (No & %)	*P-value*	ODDS RATIO& (95% CI)
Heart palpitation:				
YES	113 (33.4)	86 (24.4)	*.3826*	*1.55 (1.28 –2 1.8)*
NO	225 (66.6)	266 (75.6)		
Sleep disorder:				
YES	137 (40.5)	102 (29.0)	*.0886*	*1.67 (1.35–1.99)*
NO	201 (59.5)	250 (71.0)		
Headache:				
YES	164 (48.5)	157 (44.6)	*.2644*	*1.17 (0.82–1.52)*
NO	174 (51.5)	195 (55.4)		
Hot flushes:				
YES	196 (58.0)	136 (38.6)	*<.0001*	*2.19 (1.84–2.54)*
NO	142 (42.0)	216 (61.4)		
Irritability:				
YES	141 (41.7)	142 (40.3)	*.4852*	*1.06 (0.72–1.40)*
NO	197 (58.3)	210 (59.7)		
Joint pain:				
YES	188 (55.6)	191 (54.3)	*.7363*	*1.06 (0.72–1.40)*
NO	150 (44.4)	161 (45.7)		

Clinical characteristics findings were recorded for the study's perimenopausal and postmenopausal women (Table 3). There was no statistically significant difference in terms of waist circumference (*P=.*5284), Body mass (*P=.*6711), Height (*P=.*8659), systolic (*P=.*6724), and diastolic (0.8126) blood pressures between perimenopausal and postmenopausal women. The 2 groups had a statistically significant difference (*P<.*0001) in BMI.

**Table 3:** Clinical characteristics parameters of perimenopausal and postmenopausal women

Examination findings		Perimenopause (N=338)	Post menopause (N=352)	*P-value*
Blood pressure:				
- Systolic	Range	82–253	91–191	
	Mean	138.5	139.0	*.6724*
	SD	21.8	18.0	
- Diastolic	Range	41–146	48–112	
	Mean	80.3	80.8	*.8126*
	SD	14.4	12.7	
Height (cm):	Range	131–175	131–180	
	Mean	160	161	*.8659*
	SD	6.1	7.0	
Body mass (kg):	Range	40–184	45–143	
	Mean	80.0	85.4	
	SD	19.2	23.6	*.6711*
Waist circumference				
	Range	53–165	43 - 147	
	Mean	96.0	92.0	*.5284*
	SD	19.3	19.6	
BMI:	Range	25.5–70.1	16.3–57.6	
	Mean	28.0	26.4	*<.0001*
	SD	2.6	2.7	

BMI, Body Mass Index (kg/m^2^); BP, Blood pressure (mm Hg); waist circumference (cm).

Table 4 shows the results of biochemistry investigations carried out for the study's perimenopausal and postmenopausal women. The median triglyceride levels demonstrated an upward trend, increasing from 1.30 mmol/L in perimenopausal women to 1.99 mmol/L in postmenopausal women. The odds ratio (OR) for elevated triglycerides in the postmenopausal group was 0.65 (95% CI: 0.32–1.01). Median HDL levels rose from 1.15 mmol/L in perimenopausal women to 1.67 mmol/L in postmenopausal women. The OR of 0.83 (95% CI: 0.41–0.98) reflected a modest yet statistically significant increase in HDL concentrations after menopause. LDL levels showed the most pronounced change, with the median value more than doubling from 1.35 mmol/L in perimenopausal participants to 2.72 mmol/L in postmenopausal women. The OR of 0.50 (95% CI: 0.28–0.81) indicated a significant increase in LDL levels Postmenopause. The median AMH demonstrated a downward trend, decreasing from 2.23 ng/mL in perimenopausal to 0.6 ng/mL in postmenopausal women. The OR for lower AMH was 3.72 (95% CI: 0.21–4.10). There was no statistically significant difference in fasting glucose between perimenopausal and postmenopausal women, with a p-value of .5926.

**Table 4:** Biochemistry results of women in the study

Laboratory Investigation	Perimenopause (N=338)	Post menopause (N=352)	*Odds Ratio& (95% CI)*	*P value*
Triglycerides:				
Range	0.44–4.60	0.60–5.10	*0.65 (0.32–1.01)*	*_*
Median	1.30	1.99		
IQR	1.0–2.3	1.0–2.1		
HDL:				
Range	0.33–2.77	0.32–4.17		
Median	1.15	0.95	*0.83 (0.41–0.98)*	*_*
IQR	0.86–1.99	1.06–1.90		
LDL:				
Range	0.42–9.68	0.28–8.93		
Median	1.35	2.72	*0.50 (0.28–0.81)*	
IQR	1.22–5.26	1.17–3.70		
AMH:				*_*
Range	0.6 –8.4	0.6 -2.9		
Median	2.23	0.6	*3.72 (0.21–4.20)*	
IQR	0.67–4.92	1.08–5.32		
Fasting blood glucose				*_*
Range	4.8–18.5	3.2–18.3		
Mean	6.8	4.6	*_*	*.5926*
SD	0.6	1.2		

HDL, High Density Lipoprotein (mmol/L); LDL, Low Density Lipoprotein (mmol/L); AMH, Anti-Mullerian Hormone; IQR, Interquartile Range.

**Figure 2:**
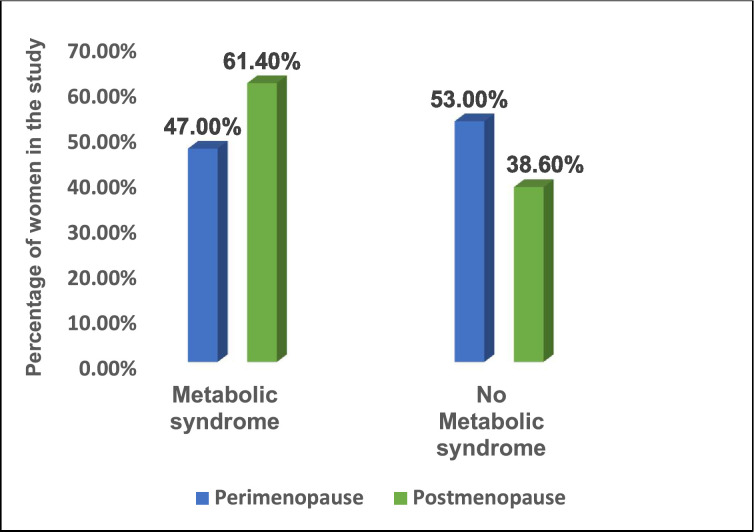
Percentage of women with metabolic syndrome (perimenopause versus post menopause).

An assessment of metabolic syndrome was evaluated for each of the 2 groups of women (perimenopausal and postmenopausal). Figure 2 below illustrates the comparison of the number of women with evidence of metabolic syndrome (peri-) versus postmenopausal women). Significantly more of the postmenopausal women (n=216; 61.4%) had evidence of metabolic syndrome as compared with perimenopausal women (n=159; 47.0%) with a statistically significant p-value of .0043.

Marapo. Metabolic syndrome in perimenopausal and postmenopausal women. Am J Obstet Gynecol 2025.

Each of the 2 groups of women was further evaluated for waist circumference, triglyceride, HDL, fasting blood glucose, and blood pressure to determine the effects of metabolic syndrome on these variables. Table 5, Table 6 show the effects of metabolic syndrome among perimenopausal and postmenopausal women in the study.

**Table 5:** Clinical profile and biochemistry variables according to the presence or absence of metabolic syndrome among perimenopausal women (n=338)

Laboratory results		Perimenopausal women with MS (N=179)	Perimenopausal women without MS (N=159)	*P-value*
Waist circumference:				
Range		53–165	55–153	
Mean		93.3	99.0	*.4882*
SD		18.1	20.1	
Triglyceride:				
Range		0.44–4.58	0.60–4.10	
Mean		1.54	1.26	*<.0001*
SD		0.70	0.52	
HDL:				
Range		0.27–2.77	0.53–2.32	
Mean		1.38	1.78	*<.0001*
SD		0.38	0.26	
Fasting blood glucose:				
Range		5.0–9.7	3.3–18.5	
Mean		6.1	5.5	*.3522*
SD		1.05	1.10	
Blood pressure:				
- systolic	Range	82–212	82–133	
	Mean	127	117	*<.0001*
	SD	18.9	11.6	
- Diastolic	Range	41–126	47–81	
	Mean	78.5	70.5	*<.0001*
	SD	12.6	7.8	

MS, Metabolic Syndrome; HDL, High Density Lipoprotein; SD, Standard Deviation.

**Table 6:** Clinical profile and biochemistry variables according to the presence or absence of metabolic syndrome among postmenopausal women (n=352)

Laboratory results		Postmenopausal women with MS (N=216)	Postmenopausal women without MS (N=136)	*P-value*
Waist circumference:				
Range		53–147	43–147	*.6723*
Mean		91.6	92.5	
SD		17.9	22.2	
Triglyceride:				
Range		0.6–4.89	0.6–5.1	*<.0001*
Mean		2.9	1.40	
SD		0.89	1.3	
			
HDL:	Range	0.32–4.17	0.24–2.80	*<.0001*
	Mean	1.29	1.70	
	SD	0.72	0.66	
Fasting blood glucose:				
Range		3.3–9.0	3.2–6.4	
Mean		4.96	4.30	*.4116*
SD		1.14	0.68	
Blood pressure:				
- systolic				
	Range	91–253	110–189	
	Mean	139.6	137.3	*.2539*
	SD	19.3	18.2	
- Diastolic	Range	48–145	49–112	
	Mean	81.3	78.9	*.3162*
	SD	14.6	12.3	

MS, Metabolic Syndrome; HDL, High Density Lipoprotein; SD, Standard Deviation.

There was a significant statistical difference in triglycerides (*P<.*0001), HDL (*P<.*0001), systolic (*P<.*0001), and diastolic (*P<.*0001) blood pressure between perimenopausal women with metabolic syndrome and those without metabolic syndrome. The 2 groups had no significant statistical difference in waist circumference (*P=.*4882) and fasting blood glucose (*P=.*3522). (Table 5).

There was a significant statistical difference in triglycerides (*P<.*0001) and HDL (*P<.*0001) between postmenopausal women with metabolic syndrome and those without metabolic syndrome. There was no statistical difference in waist circumference(*P=.*6723), fasting blood glucose (*P=.*4116), systolic (*P=.*2539), and diastolic (*P=.*3162) blood pressure between the 2 groups (Table 6).

Table 7 Multivariate logistic regression shows that age of the patients was not associated with metabolic syndrome either among perimenopausal (*P=.*3789) or post-menopausal women (*P=.*8259). Sleep disorder and heart palpitation showed statistically significant (*P<.*0001) association with metabolic syndrome among both perimenopause and post-menopausal women. Hot flushes and waist circumference were not associated with metabolic syndrome amongst perimenopausal women.

**Table 7:** Multivariate logistic regression to determine the association of age, sleep disorder, heart palpitation, waist circumference, hot flushes and joint pain with metabolic syndrome

Variables	Perimenopause				Post-menopause			
	%	Metabolic syndrome	Z-score	*P*-value	< 65 ≥ 65 (%)	Metabolic syndrome	Z-score	*P*-value
Age (yrs):								
< 45	11.4	6.1%			77%	47.7%		
≥ 45	88.6	10.6%	-0.8779	.3789	23%	49.1%	-0.2213	.8259
Sleep disorder								
yes	18.6	18.2%	+4.9618	<.001	30.7%	31.2%		<.0001
no	81.4	2.5%			69.3%	11.8%	4.3929	
Heart palpitation:								
yes	24.0	18.6%	+4.4518	<.0001	28.9%	32.4%		
no	76.0	3.8%			71.1%	9.1%	5.3212	<.0001
Hot flushes								
yes	47.9	11.8%	+0.5264	.5961	58.8%	29.4%	+2.328	.0198
no	52.1	9.2%			43.2%	18.2%		
Waist circumference								
M/S=yes	Mean 91.0	53.0%	+1.1012	.2713	Mean 96.7	60.5%	+2.256	.0114
M/S=no	Mean 95.6	47%			Mean 94.4	46.8%		

Hot flushes (*P=.*0198) and waist circumference (*P=.*0114) were significantly associated with metabolic syndrome amongst post-menopausal women.

### Comment

#### Principal findings

The prevalence of metabolic syndrome in perimenopausal and postmenopausal women was noted to be 47% and 61.4%, respectively, with a significant statistical difference between the 2 groups.

There was a significant statistical difference in age between the 2 groups, suggesting a strong correlation between age and metabolic syndrome. Perimenopausal women had significantly higher odds of experiencing hot flushes compared to postmenopausal women; these differences were statistically significant.

The 2 groups had a significant statistical difference in BMI. The mean waist circumference in perimenopausal and postmenopausal women was higher than the stipulated cut-off value of 88cm in the NCEP ATP III diagnostic criteria. However, these findings were not statistically significant, but clinically significant.

There was no significant statistical difference in terms of lipid profile between perimenopausal and postmenopausal women.

There was a significant statistical difference in HDL, triglycerides, and blood pressure between perimenopausal women with metabolic syndrome as compared with those without metabolic syndrome.

There was a significant statistical difference in HDL and triglycerides in postmenopausal women with metabolic syndrome as compared to those without.

#### Results in the context of what is known

The current study demonstrated a strong positive correlation between metabolic syndrome and age, as prevalence was higher in the postmenopausal group than in the perimenopausal group. Chedraui and co-workers (2025) noted the same findings.

The cross-sectional study conducted at Nepal Medical College Teaching Hospital from September 2023 to February 2024 by Josh A and co-workers (2024) assessed the prevalence of metabolic syndrome (MetS) among 201 perimenopausal and postmenopausal women aged 40-65. Using the International Diabetes Federation (IDF) and NCEP-ATPIII criteria, MetS prevalence was found to be 54% in perimenopausal and 61% in postmenopausal women (IDF), and 53.5% and 65%, respectively (NCEP-ATPIII). Their findings aligned with the current study's findings regarding the correlation between metabolic syndrome and age. Their findings revealed a higher percentage than the current study findings when using NCEP ATP III. The reason for this variance is attributed to the fact that their sample size was small as compared to the current study's.

The current study differs from the findings of Krakowiak J and co-workers (2022), who revealed the prevalence of MetS to be 29% in perimenopausal and 21% in postmenopausal women. Their study aimed to investigate the association between the oestrogen receptor alpha (ERα) polymorphism and the prevalence of metabolic syndrome (MetS) and obesity, as well as the coexistence of MetS and obesity in peri- and postmenopausal Polish women. Selection bias was the reason for the difference noted between the 2 studies. The current findings of perimenopausal women experiencing significantly higher odds of hot flushes as compared to postmenopausal women align with a study by Santoro N and co-workers (2015).

Crowther NJ and co-workers^13^ noted the cut-off as 91.5 cm in their study on black sub-Saharan women. Gurka and co-workers^4^ noted the waist circumference to be 90.3 cm for white Americans and 99.4cm for black Americans; both averages were higher than the cut-off point for defining abdominal obesity. Both studies are comparable to the current study findings.

Contrasting findings were noted in the study by Dorota R. and co-workers (2017), as they noted a homogenous relationship between the 2 study groups, with a p-value of .493, regarding their BMI. A study by Khouloud H and co-workers (2022) noted heterogeneity in BMI between the perimenopausal group and postmenopausal group with a p-value of <.0001, which is comparable to the current study; the difference between their findings and the current study is that their BMI was high in the postmenopausal group compared with the perimenopausal group. The current study highlights that BMI lowers in the postmenopausal period, as opposed to the high BMI noted in the postmenopausal period by other studies. These findings are a warning sign of the imminent future burden of metabolic syndrome. As this perimenopausal woman will be transitioning to the postmenopausal period, the prevalence of MetS will increase.

A study by Khouloud H. and co-workers (2022) noted a significant statistical difference in the lipid profile of perimenopausal and postmenopausal women, which differed from the current study findings. The difference is attributed to the genetic profile and socio-economic status.

#### Clinical implications

Postmenopausal women with MetS have been associated with increased risk of endometrial, ovarian, and breast cancer, as well as non-gynaecological neoplasms. Metabolic syndrome is also linked to a higher risk of uterine fibroids, which may be related to shared predisposing factors. Perimenopausal and postmenopausal women with MetS have been associated with a higher prevalence of stress urinary incontinence (SUI) when compared to those without the syndrome (Chedraui and Pérez-López, 2025).

#### Research implications

The findings in this study highlight the need for additional research, including longitudinal studies to explore causal relationships and intervention studies to assess the effectiveness of different management approaches. Sensitivity and specificity of waist circumference in defining central obesity in black African women should be assessed.

### Strength

The study's strengths include a relatively large sample size. The study followed a strict methodology adding robustness to the results found.

### Limitations

Limitations include the cohort study design, which precludes causal inferences, and the potential for selection bias due to the clinic-based recruitment. The generalizability of the findings may be limited to similar rural settings in South Africa.

## Conclusions

Study finding reflects the current high burden of metabolic syndrome amongst perimenopausal and postmenopausal women in South Africa. The prevalence of metabolic syndrome was noted to be higher in postmenopausal women as compared to perimenopausal women. There was a correlation between metabolic syndrome and age, obesity, vasomotor symptoms and dyslipidaemias.

## CRediT authorship contribution statement

**Lawrence Marapo:** Writing – review & editing, Writing – original draft, Validation, Investigation, Formal analysis, Data curation, Conceptualization. **Lineo Matsela:** Supervision. **ME Chabalala:** Writing – review & editing. **Olakunle Adewunmi Towobola:** Methodology, Formal analysis.
